# Efficacy of computer-assisted navigation in improving radiographic and clinical outcomes after total knee arthroplasty

**DOI:** 10.1097/MD.0000000000048152

**Published:** 2026-03-27

**Authors:** Wenhui Ji, Shouchao Zheng, Jing Li, Wenao Li, Han Li

**Affiliations:** a Second Department of Orthopedics, Cangzhou Hospital of Integrated Traditional Chinese and Western Medicine, Cangzhou City, Hebei Province, China; b Hebei Key Laboratory of Integrated Traditional Chinese and Western Medicine Osteoarthropathy Research, Cangzhou City, Hebei Province, China; c Department of Reproductive Medicine, Cangzhou Hospital of Integrated Traditional Chinese and Western Medicine, Cangzhou City, Hebei Province, China; d Department of Pediatric Orthopedics, Cangzhou Hospital of Integrated Traditional Chinese and Western Medicine, Cangzhou City, Hebei Province, China.

**Keywords:** component positioning, computer-assisted navigation, hip–knee–ankle angle, mechanical alignment, postoperative complications, total knee arthroplasty

## Abstract

This study aimed to compare radiographic accuracy, perioperative parameters, and early clinical outcomes between computer-assisted navigation total knee arthroplasty (TKA) and conventional TKA. This retrospective single-center study included adult patients who underwent primary TKA between January 2023 and December 2024. Patients were allocated to either a navigation group or a conventional control group based on the use of a computer-assisted navigation system. All other perioperative management followed a standardized institutional protocol. Demographic characteristics, comorbidities, and preoperative functional status – including Knee Society Score and Western Ontario and McMaster Universities Osteoarthritis Index – were comparable between groups. Primary outcomes included operative time, intraoperative blood loss, radiographic alignment parameters (hip–knee–ankle angle, femoral and tibial component positioning in coronal, sagittal, and rotational planes), and mechanical axis outliers. Secondary outcomes included early postoperative visual analog scale pain scores, knee range of motion at discharge, length of hospital stay, and complication and reoperation rates. A total of 159 patients were included, comprising 91 in the navigation group and 68 in the control group. Operative time was significantly longer in the navigation group, whereas intraoperative blood loss was significantly lower compared with the control group. Radiographic evaluation demonstrated that navigation TKA achieved more accurate restoration of the hip–knee–ankle angle, reduced mechanical axis outliers, and smaller deviations in femoral and tibial component alignment across coronal, sagittal, and rotational planes. Early postoperative clinical outcomes favored the navigation group, including lower visual analog scale pain scores, greater knee range of motion at discharge, and a modestly shorter hospital stay. Although prosthesis-related complications, medical complications, and reoperation rates were numerically lower in the navigation group, these differences did not reach statistical significance. Computer-assisted navigation TKA improved radiographic alignment accuracy and was associated with favorable early postoperative clinical outcomes compared with conventional TKA, albeit with longer operative time. While complication and reoperation rates were not significantly different, navigation may offer benefits in surgical precision and short-term recovery. Further prospective studies with long-term follow-up are warranted to determine its impact on implant survival and functional outcomes.

## 1. Introduction

Total knee arthroplasty (TKA) is an effective intervention for end-stage knee osteoarthritis and is increasingly performed in aging populations worldwide. Despite generally favorable survivorship, a substantial proportion of patients report suboptimal function, persistent pain, or dissatisfaction after surgery, which has drawn attention to technical factors such as restoration of limb alignment and accuracy of component positioning. Recent advances in computer-assisted navigation and other enabling technologies aim to improve the precision and reproducibility of TKA beyond that achievable with conventional intramedullary and extramedullary jigs.^[1,2]^ Mechanical axis restoration has long been considered a key determinant of prosthesis longevity, with deviations beyond ±3° associated with increased polyethylene wear, aseptic loosening, and revision risk in some series. Computer-assisted navigation can reduce mechanical axis outliers and improve coronal alignment of femoral and tibial components compared with conventional techniques, including in patients with substantial deformity or obesity.^[3,4]^ At the same time, evolving alignment concepts such as kinematic or restricted kinematic alignment have shifted the focus from uniform neutral alignment toward restoration of more individualized joint kinematics, often implemented through navigation or robotic platforms.^[5]^ These developments underscore the need to understand not only whether navigation improves radiographic parameters, but also how such gains relate to early pain, function, and adverse events.

Multiple meta-analyses and comparative studies have reported that computer-assisted or robotic-assisted TKA reduces mechanical axis outliers and improves component alignment, yet the magnitude of benefit in patient-reported outcomes and complication profiles remains uncertain. A recent systematic review of navigated versus conventional TKA found superior radiographic alignment with navigation, but inconsistent differences in short- to midterm clinical scores and revision rates.^[3,6]^ Similarly, meta-analyses of robotic-assisted TKA have shown improved implant positioning and a lower incidence of alignment outliers compared with conventional TKA, while functional outcomes and complication rates were largely comparable between groups.^[7,8]^ Other contemporary work has suggested that navigation and robotic technologies may provide modest advantages in early pain, range of motion (ROM), or length of stay, but not uniformly across all studies. Moreover, recent CT-based analyses highlight that a sizeable subset of patients exhibit substantial anatomical variation in the hip–knee–ankle (HKA) axis, which may complicate the application of standard mechanical alignment targets and further motivate technology-assisted, individualized planning.^[9]^

Against this background, the clinical value of computer-assisted navigation in routine primary TKA remains an area of active investigation. In particular, there is a need for studies that link detailed radiographic assessments of mechanical alignment and 3-dimensional component positioning with intraoperative parameters, early postoperative pain and function, and perioperative complications within contemporary fast-track pathways. Such evidence may help clarify the extent to which the greater technical precision afforded by navigation translates into clinically meaningful benefits and inform more rational integration of enabling technologies into standard TKA practice.

## 2. Methods

### 2.1. Study design

This study was approved by the Ethics Committee of Cangzhou Hospital of Integrated Traditional Chinese and Western Medicine. This retrospective study included adult patients diagnosed with knee osteoarthritis who underwent primary TKA at our institution between January 2023 and December 2024. Patients whose procedures were performed with the assistance of a computer-assisted navigation system were assigned to the navigation group, whereas those who underwent conventional TKA without navigation constituted the control group. All clinical and radiographic data were obtained from the institutional electronic medical record system. The study protocol adhered to the ethical principles outlined in the Declaration of Helsinki and received approval from the institutional medical ethics committee. Written informed consent was obtained from all patients prior to data collection and analysis.

Because of the retrospective observational design, allocation to the navigation or conventional group was not randomized. The choice of surgical technique was primarily determined by surgeon preference, availability of the navigation system, and patient–surgeon shared decision-making after preoperative counseling regarding potential advantages, limitations, and additional operative time associated with navigation. During the study period, both techniques were routinely used in our department. In general, no formal institutional criteria (e.g., deformity severity, body mass index [BMI] threshold, or comorbidity profile) mandated the use of navigation; however, surgeons might have been more inclined to select navigation in cases with perceived alignment challenges or complex anatomy. This nonrandom allocation introduces a potential risk of selection bias.

### 2.2. Surgical and perioperative treatment protocol

All patients underwent standardized perioperative management based on the institutional clinical pathway for TKA. Preoperative preparation included routine laboratory testing, radiographic evaluation, and anesthetic assessment to ensure surgical eligibility. Prophylactic antibiotics were administered intravenously within 30 minutes before skin incision. In the navigation group, TKA was performed using a computer-assisted navigation system to guide bone resections, restore lower-limb mechanical alignment, and optimize component positioning according to manufacturer-recommended parameters. Infrared trackers were applied following standard sterile procedures, and real-time navigation feedback was used to adjust femoral and tibial cuts, rotational alignment, and soft tissue balancing. In the control group, conventional TKA was performed using the surgeon’s standard intramedullary and extramedullary alignment instruments without navigation guidance. All surgeries were completed using cemented prostheses under tourniquet control. Postoperative care included standardized analgesia, thromboprophylaxis, early mobilization, and a structured rehabilitation program initiated within 24 hours after surgery. Radiographic assessments and functional evaluations were performed according to the institutional follow-up schedule.

### 2.3. Data collection

All data were collected retrospectively from the institutional electronic medical record system, operative reports, anesthesia charts, and the picture archiving and communication system (PACS). For each eligible patient, a standardized case report form was used to extract demographic, clinical, intraoperative, radiographic, and early postoperative outcome variables. Baseline data included age, sex, BMI, laterality of surgery, and major comorbidities, with emphasis on hypertension and diabetes mellitus. Preoperative clinical status was assessed using the Knee Society Score and the Western Ontario and McMaster Universities Osteoarthritis Index. When available, smoking status, American Society of Anesthesiologists physical status, and prior knee procedures were also recorded. Intraoperative variables were obtained from operative and anesthesia records and included procedure type (computer-assisted navigation or conventional TKA), duration of surgery, tourniquet time, estimated blood loss, and any intraoperative complications (e.g., iatrogenic fracture, ligament injury, or technical difficulties).

Radiographic data were derived from standardized postoperative long-leg weight-bearing and knee radiographs. The HKA angle was measured, and mechanical axis outliers were defined as deviations greater than ±3° from neutral. For femoral and tibial components, coronal and sagittal alignment were recorded as absolute deviations from target angles, and rotational alignment was assessed using established anatomical references. All measurements were performed in PACS by experienced orthopedic surgeons according to a predefined protocol. Early postoperative clinical outcomes were obtained from inpatient charts and discharge summaries. Pain intensity was evaluated using the visual analog scale (VAS, 0–10) on postoperative days 1 and 3 and at discharge. Knee ROM at discharge and length of hospital stay (days from surgery to discharge) were also recorded. Postoperative complications and adverse events were identified from inpatient documentation, early follow-up records, and readmission notes. Prosthesis-related complications included malalignment, residual instability, and suspected aseptic loosening. Medical complications comprised surgical site infection, thromboembolic events, and clinically relevant stiffness. Reoperations related to the index TKA were documented separately.

To enhance measurement reliability, all radiographic parameters were independently assessed by 2 experienced orthopedic surgeons who were not involved in the index surgery. Measurements were performed using standardized PACS tools according to a predefined protocol. Observers were blinded to the surgical technique used for each patient at the time of measurement. Inter-observer reliability was assessed using the intraclass correlation coefficient. For parameters with discrepant measurements >2°, consensus was reached through joint review.

### 2.4. Statistical analysis

Statistical analyses were performed using IBM SPSS Statistics, version 26.0 (IBM Corp., Armonk). Continuous variables (e.g., age, BMI, operative time, blood loss, alignment parameters, VAS scores, ROM, length of hospital stay) were expressed as mean ± standard deviation and compared between the navigation and control groups using independent samples *t* tests. Categorical variables (e.g., sex, comorbidities, proportion of mechanical axis outliers, intraoperative and postoperative complications, reoperation rates) were presented as counts and percentages and compared using the chi-square test. All statistical tests were two-sided, and a *P*-value < .05 was considered statistically significant. Given the moderate sample size and the relatively low incidence of postoperative complications and reoperations, multivariate regression analysis was not performed.

## 3. Results

### 3.1. Patient enrollment and baseline characteristics

A total of 175 patients with knee osteoarthritis who underwent primary TKA between January 2023 and December 2024 were screened. After applying the inclusion and exclusion criteria, 159 patients were included in the final analysis, with 68 patients in the control group (conventional TKA) and 91 patients in the navigation group (computer-assisted navigation TKA). Baseline demographic and clinical characteristics are summarized in Table 1. There were no statistically significant differences between the 2 groups for any baseline variable. The mean age was 69.5 ± 6.2 years in the control group and 68.9 ± 6.8 years in the navigation group (*t* = 0.58, *P* = .563). The proportion of male patients was 47.1% (32/68) in the control group and 41.8% (38/91) in the navigation group (χ^2^ = 0.44, *P* = .505). BMI was comparable between groups (27.5 ± 2.9 kg/m^2^ vs 27.9 ± 2.7 kg/m^2^; *t* = −0.89, *P* = .377). The distribution of laterality was also similar, with right knees accounting for 64.7% (44/68) of procedures in the control group and 65.9% (60/91) in the navigation group (χ^2^ = 0.03, *P* = .872).

**Table 1:** Baseline characteristics of patients undergoing total knee arthroplasty.

Variable	Control group (n = 68)	Navigation group (n = 91)	Test statistic	*P*-value
Age, yr	69.5 ± 6.2	68.9 ± 6.8	*t* = 0.58	.563
Male, n (%)	32 (47.1)	38 (41.8)	χ^2^ = 0.44	.505
BMI, kg/m^2^	27.5 ± 2.9	27.9 ± 2.7	*t* = −0.89	.377
Right knee, n (%)	44 (64.7)	60 (65.9)	χ^2^ = 0.03	.872
Diabetes mellitus, n (%)	14 (20.6)	21 (23.1)	χ^2^ = 0.14	.708
Hypertension, n (%)	40 (58.8)	54 (59.3)	χ^2^ = 0.00	.948
Preoperative KSS	48.2 ± 8.6	49.7 ± 8.1	*t* = −1.12	.267
Preoperative WOMAC	60.5 ± 10.3	59.1 ± 11.8	*t* = 0.80	.427

BMI = body mass index, KSS = Knee Society Score, WOMAC = Western Ontario and McMaster Universities Osteoarthritis Index.

The prevalence of diabetes mellitus was 20.6% (14/68) in the control group and 23.1% (21/91) in the navigation group (χ^2^ = 0.14, *P* = .708), while hypertension was present in 58.8% (40/68) and 59.3% (54/91) of patients, respectively (χ^2^ = 0.00, *P* = .948). Preoperative functional status did not differ significantly between groups: the mean preoperative Knee Society Score was 48.2 ± 8.6 in the control group and 49.7 ± 8.1 in the navigation group (*t* = −1.12, *P* = .267), and the corresponding Western Ontario and McMaster Universities Osteoarthritis Index scores were 60.5 ± 10.3 and 59.1 ± 11.8, respectively (*t* = 0.80, *P* = .427). Overall, the 2 groups were well balanced with respect to demographic characteristics, comorbidities, and preoperative functional scores (Table 1).

### 3.2. Intraoperative parameters

The mean duration of surgery was significantly longer in the navigation group than in the control group (95.0 ± 14.0 minutes vs 85.0 ± 12.0 minutes; *t* = 4.84, *P* < .001). Intraoperative blood loss was lower in the navigation group compared with the control group (230.0 ± 75.0 mL vs 260.0 ± 80.0 mL; *t* = 2.40, *P* = .018). Tourniquet time showed no statistically significant difference between the 2 groups (62.0 ± 11.0 minutes in the navigation group vs 65.0 ± 10.0 minutes in the control group; *t* = 1.79, *P* = .075). Intraoperative complications occurred in 5 of 68 patients (7.4%) in the control group and in 4 of 91 patients (4.4%) in the navigation group, and this difference was not statistically significant (χ^2^ = 0.64, *P* = .425; Table 2).

**Table 2:** Intraoperative parameters in patients undergoing total knee arthroplasty.

Variable	Control group (n = 68)	Navigation group (n = 91)	Test statistic	*P*-value
Duration of surgery, min	85.0 ± 12.0	95.0 ± 14.0	*t* = 4.84	<.001
Intraoperative blood loss, mL	260.0 ± 80.0	230.0 ± 75.0	*t* = 2.40	.018
Tourniquet time, min	65.0 ± 10.0	62.0 ± 11.0	*t* = 1.79	.075
Intraoperative complications, n (%)	5 (7.4)	4 (4.4)	χ^2^ = 0.64	.425

mL = milliliter, min = minutes.

### 3.3. Radiographic outcomes

Postoperative radiographic parameters are summarized in Table 3. The mean postoperative HKA angle was 179.1° ± 2.6° in the control group and 180.0° ± 1.9° in the navigation group, indicating closer restoration to neutral mechanical alignment in the navigation group (*t* = −2.41, *P* = .017). The proportion of mechanical axis outliers, defined as an HKA deviation greater than ±3° from neutral, was 25.0% (17/68) in the control group and 8.8% (8/91) in the navigation group (χ^2^ = 7.72, *P* = .005). With respect to component positioning, all alignment variables are expressed as the absolute deviation from the planned target angle. The femoral component coronal deviation was 1.9° ± 1.1° in the control group and 1.3° ± 0.8° in the navigation group (*t* = 3.81, *P* < .001), while the tibial component coronal deviation was 2.1° ± 1.2° and 1.4° ± 0.9°, respectively (*t* = 4.04, *P* < .001). In the sagittal plane, femoral component deviation was 2.0° ± 1.3° in the control group versus 1.5° ± 1.0° in the navigation group (*t* = 2.64, *P* = .009), and tibial slope deviation was 2.3° ± 1.4° vs 1.7° ± 1.1° (*t* = 2.92, *P* = .004). Rotational alignment also favored the navigation group: femoral rotational deviation was 2.6° ± 1.5° in the control group and 1.8° ± 1.2° in the navigation group (*t* = 3.62, *P* < .001), and tibial rotational deviation was 3.0° ± 1.8° vs 2.1° ± 1.4° (*t* = 3.42, *P* < .001). Overall, computer-assisted navigation reduced both mean deviation and variability of mechanical alignment and component positioning compared with conventional TKA (Table 3).

**Table 3:** Postoperative radiographic outcomes after total knee arthroplasty.

Variable	Control group (n = 68)	Navigation group (n = 91)	Test statistic	*P*-value
Postoperative HKA angle, °	179.1 ± 2.6	180.0 ± 1.9	*t* = −2.41	.017
HKA outliers >±3°, n (%)	17 (25.0)	8 (8.8)	χ^2^ = 7.72	.005
Femoral component coronal deviation, °	1.9 ± 1.1	1.3 ± 0.8	*t* = 3.81	<.001
Tibial component coronal deviation, °	2.1 ± 1.2	1.4 ± 0.9	*t* = 4.04	<.001
Femoral component sagittal deviation, °	2.0 ± 1.3	1.5 ± 1.0	*t* = 2.64	.009
Tibial component sagittal (slope) deviation, °	2.3 ± 1.4	1.7 ± 1.1	*t* = 2.92	.004
Femoral component rotational deviation, °	2.6 ± 1.5	1.8 ± 1.2	*t* = 3.62	<.001
Tibial component rotational deviation, °	3.0 ± 1.8	2.1 ± 1.4	*t* = 3.42	<.001

° = degrees, HKA = hip–knee–ankle.

### 3.4. Early postoperative clinical outcomes

Pain intensity assessed by the VAS (0–10) was lower in the navigation group than in the control group at all recorded time points. On postoperative day 1, the mean VAS score was 5.8 ± 1.1 in the control group and 5.2 ± 1.0 in the navigation group (*t* = 3.54, *P* < .001). On postoperative day 3, VAS scores were 4.2 ± 1.0 versus 3.7 ± 0.9, respectively (*t* = 3.25, *P* = .001). At discharge, VAS scores decreased to 3.0 ± 0.9 in the control group and 2.6 ± 0.8 in the navigation group (*t* = 2.91, *P* = .004). Knee ROM at discharge was higher in the navigation group (100° ± 11°) than in the control group (95° ± 10°), and the difference was statistically significant (*t* = −2.99, *P* = .003). Length of hospital stay was slightly shorter in the navigation group (7.4 ± 1.5 days) compared with the control group (8.0 ± 1.6 days; *t* = 2.40, *P* = .017; Table 4).

**Table 4:** Early postoperative clinical outcomes after total knee arthroplasty.

Variable	Control group (n = 68)	Navigation group (n = 91)	Test statistic	*P*-value
VAS score, d 1 (0–10)	5.8 ± 1.1	5.2 ± 1.0	*t* = 3.54	<.001
VAS score, d 3 (0–10)	4.2 ± 1.0	3.7 ± 0.9	*t* = 3.25	.001
VAS score at discharge (0–10)	3.0 ± 0.9	2.6 ± 0.8	*t* = 2.91	.004
ROM at discharge, °	95 ± 10	100 ± 11	*t* = −2.99	.003
Length of hospital stay, d	8.0 ± 1.6	7.4 ± 1.5	*t* = 2.40	.017

ROM = range of motion, VAS = visual analog scale.

### 3.5. Postoperative complications and adverse events

Prosthesis-related complications, including malalignment, residual instability, or radiographic suspicion of aseptic loosening, occurred in 5 of 68 patients (7.4%) in the control group and 3 of 91 patients (3.3%) in the navigation group. The difference between groups was not statistically significant (χ^2^ = 1.340, *P* = .247). Medical complications, defined as postoperative infection (superficial or deep), thromboembolic events, or clinically relevant stiffness requiring intensified rehabilitation, were observed in 12 patients (17.6%) in the control group and 8 patients (8.8%) in the navigation group (χ^2^ = 2.776, *P* = .096). Although the navigation group showed a numerically lower rate of medical complications, the difference did not reach statistical significance. Reoperations for any cause during the observation period were required in 4 patients (5.9%) in the control group and 2 patients (2.2%) in the navigation group (χ^2^ = 1.455, *P* = .228). Overall, the navigation group showed consistently lower rates of prosthesis-related and medical complications and reoperations, but no between-group difference reached statistical significance (Table 5).

**Table 5:** Postoperative complications and adverse events after total knee arthroplasty.

Variable	Control group (n = 68)	Navigation group (n = 91)	Test statistic	*P*-value
Prosthesis-related complications, n (%)	5 (7.4)	3 (3.3)	χ^2^ = 1.340	.247
Medical complications, n (%)	12 (17.6)	8 (8.8)	χ^2^ = 2.776	.096
Reoperations, n (%)	4 (5.9)	2 (2.2)	χ^2^ = 1.455	.228

## 4. Discussion

This retrospective study evaluated the impact of computer-assisted navigation on intraoperative parameters, radiographic alignment, early postoperative recovery, and short-term complications after TKA. The principal findings were that navigation was associated with a longer operative time but reduced intraoperative blood loss, significantly improved restoration of the mechanical axis and component positioning in all planes, modest but consistent improvements in early pain and functional recovery, and numerically lower rates of prosthesis-related and medical complications without statistically significant between-group differences.

The prolongation of operative time in the navigation group is consistent with previous reports indicating that computer-assisted techniques require additional steps for tracker placement, registration, and intraoperative verification. Recent work on kinematic navigation similarly reported increased operative duration without device-related technical failures.^[10]^ However, the reduction in intraoperative blood loss observed in the present study suggests that navigation may facilitate more controlled bone cuts and avoid intramedullary canal violation, thereby limiting bleeding. A recent meta-analysis focusing on NAVIO-assisted TKA reported improved alignment with a small but significant reduction in blood loss relative to conventional TKA, which is in line with these findings.^[6]^ The absence of a difference in tourniquet time indicates that the additional time required for navigation was likely distributed across non-tourniquet phases or compensated by more efficient execution of bone resections once registration was completed.

From a radiographic standpoint, the navigation group showed closer restoration of the HKA angle to neutral and a significantly lower proportion of mechanical axis outliers beyond ±3°, together with smaller deviations in femoral and tibial component alignment in coronal, sagittal, and rotational planes. These data support the concept that navigation can improve both accuracy and precision of implant positioning compared with conventional instrumentation. Recent meta-analyses and systematic reviews have confirmed that computer-assisted TKA consistently reduces mechanical axis outliers and improves coronal alignment of both femoral and tibial components relative to conventional TKA.^[3]^ In a 2024 systematic review focusing on computer-assisted navigation in complex deformity, CAS-TKA achieved acceptable mechanical axis alignment with mean deviations close to neutral, even in cases with extra-articular deformity.^[11]^ Similarly, a contemporary review on navigation in TKA concluded that computer-assisted techniques reliably improve alignment metrics, although translation of these gains into long-term clinical benefit remains variable. The radiographic improvements demonstrated in the present cohort, therefore, align with the prevailing evidence that navigation offers measurable advantages in alignment control.

The extent to which improved alignment translates into better clinical outcomes has been debated. In this study, the navigation group had lower pain scores on postoperative days 1 and 3 and at discharge, greater ROM at discharge, and a modest reduction in length of hospital stay. These findings suggest that more accurate restoration of limb alignment and joint kinematics may facilitate early rehabilitation and functional recovery. A recent analysis of the relationship between femoral component alignment and short-term outcomes indicated that deviations in alignment were associated with inferior functional scores and patient satisfaction after TKA, supporting the clinical relevance of precise component positioning.^[12]^ Moreover, recent comparative studies of advanced technologies such as robotic or accelerometer-based navigation have reported improved early functional recovery and shorter hospital stay, even when mid-term clinical scores are similar between groups.^[13]^ The present findings are compatible with these reports, indicating that technology-assisted techniques, including navigation, may confer advantages during the early postoperative period, particularly in pain control and mobilization.

However, the absence of a statistically significant reduction in early prosthesis-related and medical complications or reoperations is also consistent with recent literature. A 2023 comparative analysis of computer-assisted versus conventional mechanically aligned TKA found no clear advantage of navigation in terms of short-term complications or functional scores, despite improved radiographic alignment.^[14]^ Likewise, a recent long-term follow-up study of kinematic navigation reported better alignment but no improvement in implant survival or functional outcomes compared with conventional TKA. These observations suggest that while navigation enhances technical precision, its effect on early complication rates and long-term survivorship may be modest, and may become apparent only in specific subgroups (e.g., severe deformity, extra-articular malalignment, or high functional demand). The nonsignificant trends toward fewer prosthesis-related and medical complications in the navigation group in this study may therefore reflect an underpowered comparison for relatively infrequent adverse events rather than the absence of any true difference.

Taken together, the current data support a model in which computer-assisted navigation improves mechanical alignment and component positioning and is associated with modest benefits in early pain and function, while its impact on short-term complications remains limited. The clinical significance of reducing mechanical axis outliers should also be considered in the context of evolving alignment philosophies. While traditional mechanical alignment targets strict neutral alignment, recent work has explored kinematic or functional alignment strategies that prioritize restoration of patient-specific joint kinematics and may tolerate small deviations from neutral.^[15]^ In this framework, navigation could be used not only to enforce neutral mechanical alignment but also to reproduce individualized alignment targets in a reproducible manner. From a clinical perspective, the findings indicate that computer-assisted navigation can be a valuable adjunct when the primary objective is to optimize radiographic alignment and enhance early recovery after TKA. The observed reductions in pain and hospital stay, together with improved ROM at discharge, suggest that navigation may contribute to more efficient rehabilitation pathways and potentially lower early resource utilization, particularly in institutions with standardized perioperative care and early mobilization protocols. At the same time, the lack of a clear reduction in complication rates and the requirement for additional operative time highlight that navigation should be integrated selectively, with consideration of patient characteristics, surgeon experience, and institutional workflow.

First, the non-randomized retrospective design introduces the possibility of selection bias. Although baseline demographic and preoperative clinical variables were statistically comparable between groups, unmeasured confounders – such as deformity complexity, bone quality, or surgeon-specific preferences – may have influenced the choice of technique. If navigation was preferentially used in more complex cases, the observed radiographic advantages may be conservative estimates; conversely, if it was selected for technically straightforward cases, the magnitude of benefit may be overestimated. Therefore, causality cannot be definitively established. Second, multivariate regression analysis was not conducted. Although baseline characteristics were comparable, residual confounding cannot be excluded. The relatively limited sample size and low frequency of adverse events restricted the feasibility of robust multivariable modeling. As a result, the associations observed between navigation use and early outcomes should be interpreted as exploratory rather than definitive. Future larger, preferably prospective studies should incorporate multivariate adjustment or propensity score matching to better control for confounding factors.

## 5. Conclusion

In this retrospective cohort, computer-assisted navigation in TKA improved restoration of mechanical alignment and component positioning and was associated with reduced intraoperative blood loss, lower early postoperative pain, greater ROM at discharge, and slightly shorter hospital stay, at the cost of increased operative time. These findings are limited to the early postoperative period and do not imply superior long-term implant survivorship or sustained functional advantages. Further prospective studies with long-term follow-up are required to determine whether the observed radiographic improvements translate into durable clinical benefits.

## Author contributions

**Conceptualization:** Wenhui Ji, Shouchao Zheng, Jing Li, Wenao Li, Han Li.

**Funding acquisition:** Wenhui Ji, Jing Li.

**Data curation:** Wenhui Ji, Shouchao Zheng, Jing Li, Wenao Li, Han Li.

**Formal analysis:** Wenhui Ji, Shouchao Zheng, Jing Li, Wenao Li, Han Li.

**Investigation:** Jing Li.

**Writing – original draft:** Wenhui Ji, Jing Li.

**Writing – review & editing:** Wenhui Ji, Jing Li.
